# Deciphering evolution of immune recognition in antibodies

**DOI:** 10.1186/s12900-018-0096-1

**Published:** 2018-12-19

**Authors:** Harmeet Kaur, Neetu Sain, Debasisa Mohanty, Dinakar M. Salunke

**Affiliations:** 1 Regional Centre for Biotechnology, Biotech Science Cluster, Faridabad, Haryana 121001 India; 2 Manipal Academy of Higher Education, Manipal, Karnataka 576104 India; 3 National Institute of Immunology, New Delhi, Delhi 110067 India; 4 International Centre for Genetic Engineering and Biotechnology, New Delhi, Delhi 110067 India

**Keywords:** Germline, Mature, Somatic hypermutation, Data science, Antigens, Paratope, Antibody, Simulation, Cluster, Conformation

## Abstract

**Background:**

Antibody, the primary effector molecule of the immune system, evolves after initial encounter with the antigen from a precursor form to a mature one to effectively deal with the antigen. Antibodies of a lineage diverge through antigen-directed isolated pathways of maturation to exhibit distinct recognition potential. In the context of evolution in immune recognition, diversity of antigen cannot be ignored. While there are reports on antibody lineage, structural perspective with respect to diverse recognition potential in a lineage has never been studied. Hence, it is crucial to evaluate how maturation leads to topological tailoring within a lineage enabling them to interact with significantly distinct antigens.

**Results:**

A data-driven approach was undertaken for the study. Global experimental mouse and human antibody-antigen complex structures from PDB were compiled into a coherent database of germline-linked antibodies bound with distinct antigens. Structural analysis of all lineages showed variations in CDRs of both H and L chains. Observations of conformational adaptation made from analysis of static structures were further evaluated by characterizing dynamics of interaction in two lineages, mouse *V*_*H*_*1–84* and human *V*_*H*_*5–51*. Sequence and structure analysis of the lineages explained that somatic mutations altered the geometries of individual antibodies with common structural constraints in some CDRs. Additionally, conformational landscape obtained from molecular dynamics simulations revealed that incoming pathogen led to further conformational divergence in the paratope (as observed across datasets) even while maintaining similar overall backbone topology. MM-GB/SA analysis showed binding energies to be in physiological range. Results of the study are coherent with experimental observations.

**Conclusions:**

The findings of this study highlight basic structural principles shaping the molecular evolution of a lineage for significantly diverse antigens. Antibodies of a lineage follow different developmental pathways while preserving the imprint of the germline. From the study, it can be generalized that structural diversification of the paratope is an outcome of natural selection of a conformation from an available ensemble, which is further optimized for antigen interaction. The study establishes that starting from a common lineage, antibodies can mature to recognize a wide range of antigens. This hypothesis can be further tested and validated experimentally.

**Electronic supplementary material:**

The online version of this article (10.1186/s12900-018-0096-1) contains supplementary material, which is available to authorized users.

## Background

The antibody-antigen (Ab-Ag) is a miniature system to understand the process of evolution. Development of a B-cell starting from progenitor lymphoid cell to an immature B-cell marks the event of VDJ recombination leading to the formation of naïve germline antibody (Ab) [1]. Exposure of an antigen (Ag) fosters affinity maturation leading to an iterative process of cell proliferation, extensive mutagenesis on the immunoglobulin gene and stringent Darwinian selection of B-cells producing higher affinity antibodies [2]. Various studies have also reported insertions and deletions during somatic hypermutation [3, 4]. Alterations in the genetic machinery of immunoglobulin are tailored to mediate antigen recognition. Such genetic drifts are strictly dependent on the time of exposure and antigen persistence [5]. Hence, at any given time, the incoming antigen acts as a stimulus that allows somatic mutations to be incorporated in the genome that may be favorable and rarely deleterious. Infrequent deleterious mutations result in formation of self-reactive receptors or B-cell transformation [6]. These outcomes are generally eliminated by regulated cell-cycle checkpoints and negative selection processes in the germinal center. Nonetheless, the processes often undergo challenge that may result in breach of self-tolerance leading to autoimmune disorders. Favorable mutations, on the other hand, undergo affinity-based positive selection and facilitate recognition of bona fide antigen. Therefore, based on the incoming antigen, different pathways of maturation may ensue in the germline B-cells forming different advanced versions or siblings of a lineage. Detailed characterization of dynamics of antigen recognition by mature antibodies should provide unprecedented insight into the immune response. Thus, an Ab-Ag system serves as a perfect miniature model to study the evolution of molecular recognition i.e. how antibodies of an ontology diffuse into isolated molecular environment of the stimulating antigen enabling differentiated fixation of paratope topology. One way of dissecting this is by looking for structural changes in the lineage. While sequence based phylogeny has been implemented to analyze different facets of evolution of immunological recognition, the role of structural adaptation in a lineage has not been systematically explored.

Studies on antibodies inheriting the same set of germline genes suggest adoption of fundamentally different binding modes [7]. In the present study, we have examined conformational features associated with recognition of a wide range of antigens by sibling antibodies. Additionally, it is hypothesized that any similarity despite divergence in the structural landscape obtained from simulation will explain their common origin.

Towards this end, structural data of antibodies of different lineages bound with significantly distinct antigens were collated and analyzed. Topological alteration of the CDRs, to accommodate distinct antigens was evident within individual lineage. To validate observation from analysis of static structures, antibodies of two germline lineages i.e. carrying the same *V*_*H*_ gene set and bound to significantly different antigens were analyzed separately using explicit solvent molecular dynamics (MD) simulations. While sequence analysis suggested that somatic mutations have altered the geometry of the respective antibody, dynamics showcased that further structural divergence specifically in the paratope was brought about for preferential antigen binding. Despite somatic diversification, similar overall architecture of the descendants and the conformers could be envisaged as retention of germline imprint.

## Results

### Germline-linked mature antibodies reveal structural heterogeneity

**Fig. 1:**
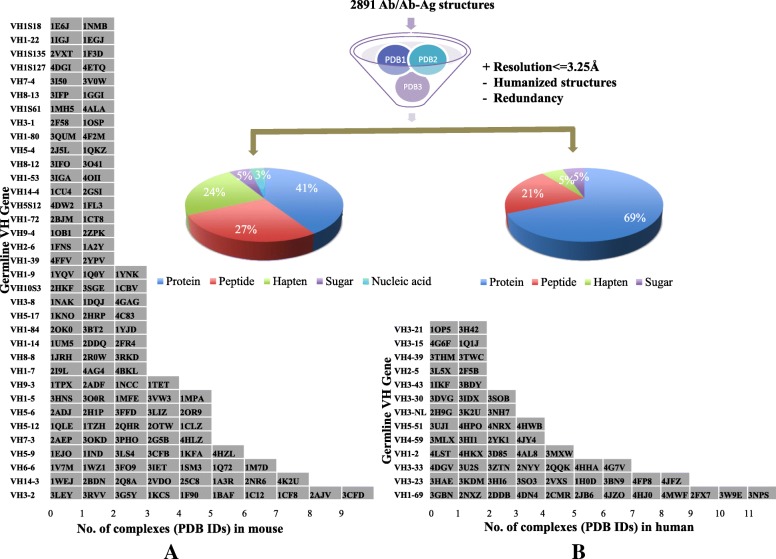
Data collation. Compiled dataset of Ab-Ag complexes from (**a**) mouse and (**b**) human, retrieved from PDB**.** Antibodies sharing common germline *V*_*H*_ gene (shown along Y-axis) were clustered together and the number of complexes is plotted along X-axis with their PDB IDs. Percentage of chemically distinct antigens bound by mature antibodies is represented in the pie chart

Two thousand eight hundred ninety-one hits were obtained from RCSB PDB that contained both standalone antibody molecules and Ab-Ag complexes [8]. Coordinate files of complexes were retrieved and were segregated based on the source of antibody into two groups, mouse and human, to obtain homogenous sets of data. The hits were filtered based on the following criteria: Only X-ray crystallographic structures with resolution of 3.25 Å or higher were retained. Humanized or chimeric structures were excluded, only a single representative of an antibody was taken to avoid redundancy. Criteria for the nature of antigen or antibody isotype was not set. Antibodies that are deemed to share the same lineage are those that are produced from a single B-cell clone. It has been documented that the H-chain is the primary player in antigen recognition and binding [9, 10]. The L-chain, although, acts in concert with H-chain, is largely known to be an accessory component. Therefore, at the molecular level, the data were subsequently clustered based on a recent study that involved antibody evolution from a common ancestral *V*_*H*_ (variable region of heavy chain) gene [11]. The coding genes of retrieved antibodies were identified based on highest score and lowest e-value. If the antibodies aligned with similar scores to several distinct germline genes, the gene that was also common in other antibodies and enabled their clustering was selected. Those that shared no common IGHV origin were rejected. The final compiled dataset comprised of 115 mature antibodies from mouse that were grouped into 35 clusters based on common germline *V*_*H*_ genes (Fig. 1a and Additional file 1: Table S1). Likewise, 57 mature antibodies from human were clustered into 13 germline *V*_*H*_ gene groups (Fig. 1b and Additional file 2: Table S2).

In the collated database, the chemistry of antigens bound by antibodies evolved from a common IGHV was analyzed. The data revealed a high level of diversity and individual uniqueness of the antigens (Fig. 1; Additional file 1: Table S1 and Additional file 2: Table S2). Antigens of individual sets could not be aligned because of distinctness in their chemical nature and topology. Even for protein or peptide antigens, sequence alignment revealed no immuno-dominant epitopes as seen for anti-peptide antibodies of *V*_*H*_*1S127, V*_*H*_*1–53, V*_*H*_*1–39, V*_*H*_*1–80* etc. lineages from mouse and *V*_*H*_*3–30, V*_*H*_*1–2, V*_*H*_*3–33, V*_*H*_*5–51* etc. lineages from human (Additional file 1: Table S1 and Additional file 2: Table S2). Chemically, the spectrum of epitopes was wide for mouse *V*_*H*_*3–2, V*_*H*_*7–3, V*_*H*_*1–84, V*_*H*_*1–5* and *V*_*H*_*5–17* lineages and for human *V*_*H*_*1–69, V*_*H*_*3–23, V*_*H*_*4–59* and *V*_*H*_*4–39* lineages ranging from proteins, peptides, nucleic acid to sugar and haptens.

**Fig. 2:**
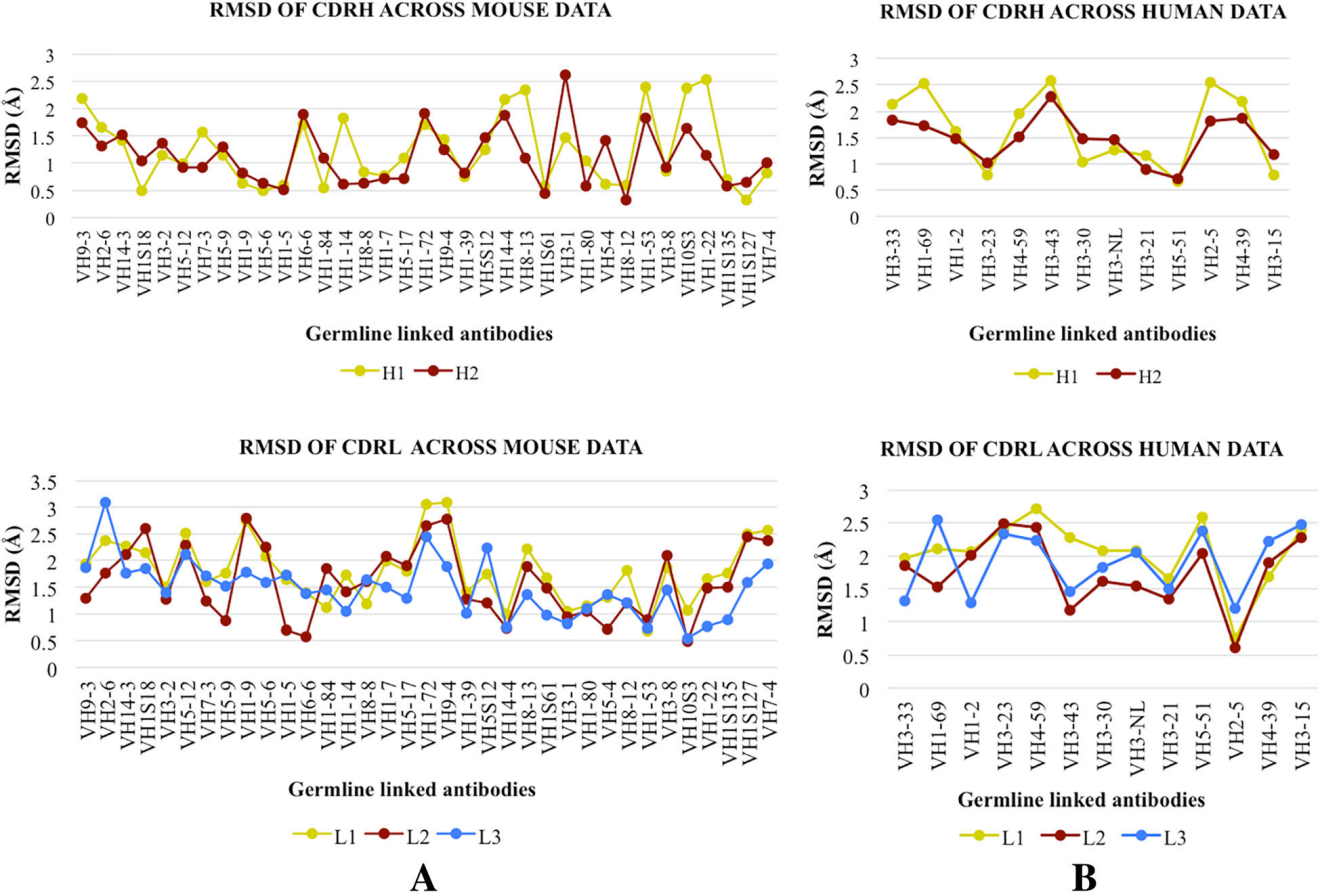
Variability in CDR loops of antibodies. RMSD plots of CDR loops of heavy and light chains of antibodies from (**a**) mouse and (**b**) human

Despite significant diversity in antigens, it was intriguing that they were bound by sibling antibodies. Structure superposition of all antibodies of respective lineages was done to check topological changes undergone in CDRs to accommodate respective antigens. Conformational variability of the paratope was apparent for all groups of mouse and human (Additional file 3: Figures S1 and S2). Variability was observed in all CDR loops of both heavy and light chains. However, the contribution of individual CDR loop was variable for different datasets. To further quantify this observation, average RMSDs of CDR loops were calculated from structural alignment. In case of mouse, 58 and 52% data show RMSD > 1 Å for CDRs H1 and H2 respectively while 98, 80 and 80.5% data show RMSD > 1 Å for CDRs L1, L2 and L3 respectively (Fig. 2a). In case of human, 76 and 84% data had RMSD > 1 Å for CDRs H1, H2 while 92, 92 and 100% data for CDRs L1, L2 and L3 (Fig. 2b). Due to recombination events at V-D and D-J junctions [12], the sequence and length of H3 is significantly altered within a lineage across dataset, hence its RMSD could not be computed. Thus, from analysis of structural alignment it is clear that all CDR loops exhibit seemingly comparable variability; the RMSD values being slightly lower for H1 and H2 in mouse antibodies. The structural alignment provides a basic understanding of the conformational re-arrangement that facilitates a favorable micro-environment for accommodating the antigen. It does not necessarily translate into actual contacts made by CDRs with the antigen.

In order to further assess contributions of CDR loops, interaction profile of the complexes in the light of H-bond formation was investigated. Engagement of H-chain was higher than L-chain in 71% mouse and 78% human data, with contributions of all three loops (Additional file 3: Figures S3 and S4). 14% (mouse) and 24.5% (human) data show no involvement of L-chain. In 12% mouse (PDB 1TPX of *V*_*H*_*9–3*, 1E6J of *V*_*H*_*1S18*, 1BAF of *V*_*H*_*3–2* etc.) and 1% human (PDB 4DGV of *V*_*H*_*3–33*, 2JB6 of *V*_*H*_*1–69*, 4JFZ of *V*_*H*_*3–23* etc.) data, H3 does not form any H-bond, implying significant contributions of CDRs H1 and H2. But, H3, in conjunction with other loops, is crucial for antigen recognition and binding as seen across rest of the data [10]. In general, H3 plays a predominant role in defining the topography of the binding site [13]. Shorter ones can create a cavity to accommodate peptides while long H3 loops can generate a definite finger-like topography [14, 15]. Thus, structural re-arrangement in all CDRs of the collated dataset and especially in H-chain that bear common genetic elements was surprising.

Thus, sibling antibodies exhibit conformational heterogeneity of the paratope to accommodate distinct antigens and interaction is mediated by different CDR loops with contribution of varying degrees across dataset. Even though H3 is a crucial player in antigen recognition, it may not necessarily be involved in direct contact with the antigen (as observed from H-bond analysis), signifying the involvement of other loops. The appropriately screened database thus served as an ideal resource to understand divergent evolution. However, mechanistic details would be better revealed by investigating dynamics of a lineage as it would shed light on time-dependent structural changes.

Towards this direction, two lineages were chosen from the compiled datasets, *V*_*H*_*5–51* from human and *V*_*H*_*1–84* from mouse (Table 1, for details, see Methods). Important requisites for selection of systems were that antigens bound by sibling antibodies were different and forcefield parameters for antigens were available for subsequent simulation studies or could be generated using available tools. The sequences and structures of these antigens were analyzed to ensure they were different.

**Table 1:** Ab-Ag complexes as test systems for study

Organism	PDB ID	Antibody	Antigen	*V*_*H*_-gene	Score (Bits)	e-value
Mouse	2OK0	ED10	DNA	1–84*01	171	2e-44
	3BT2	Anti-uPAR	Urokinase plasminogen activator surface receptor, Vitronectin	1–84*01	162	1e-41
	1YJD	5.11A1	T-cell-specific surface glycoprotein CD28	1–84*01	140	5e-35
Human	4NRX	m66	Gp41 MPER peptide	5–51*03	219	5e-59
	4HWB	10G5H6	Ectodomain D3 of IL-13	5–51*03	237	3e-64
	3UJI	2558	Gp160 V3-domain	5–51*03	204	2e-54
	4HPO	CH58	Gp120 V2-domain	5–51*03	243	5e-66

### Mapping somatic mutations in mature antibodies

**Fig. 3:**
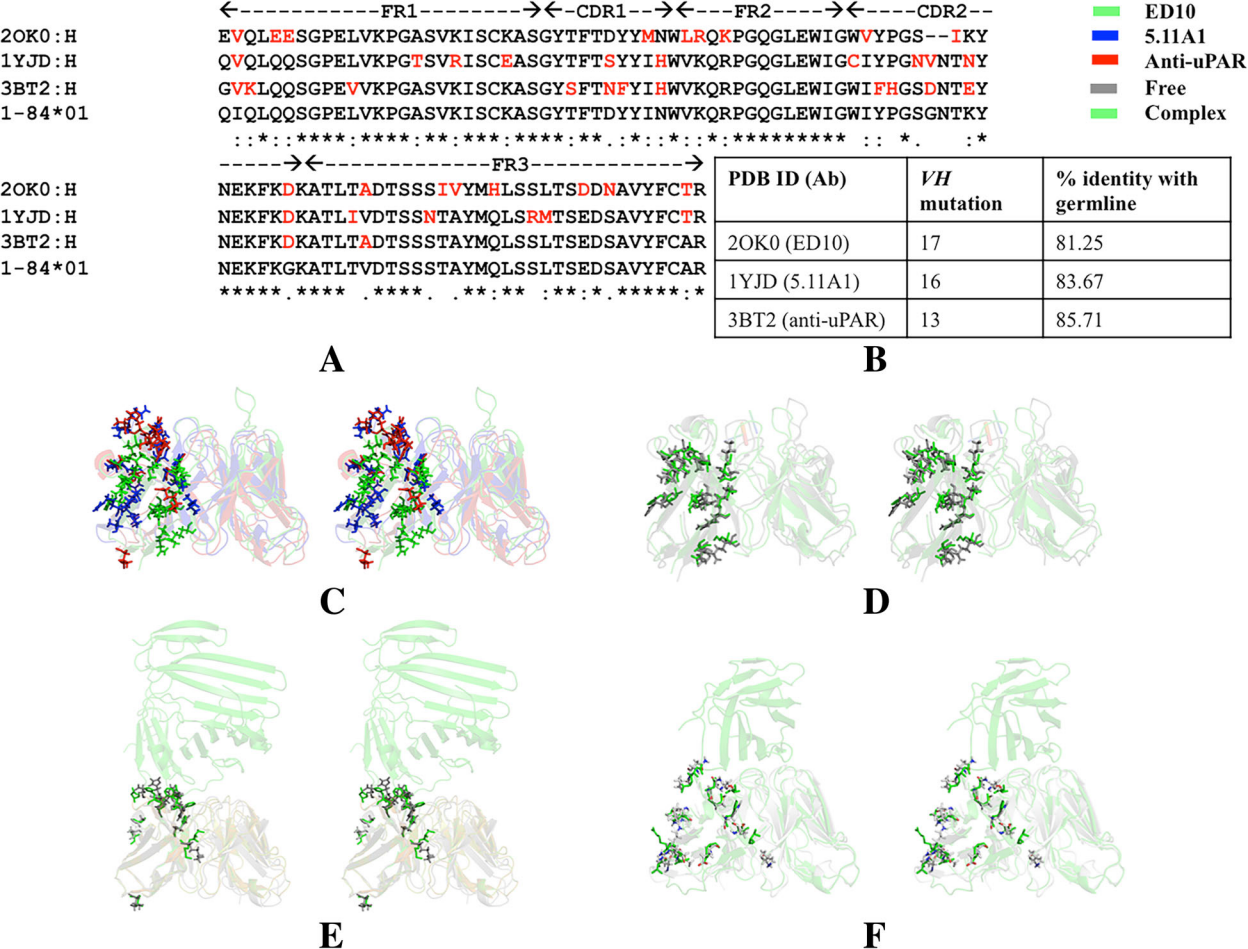
Mapping somatic mutations on sequences and structures of antibodies of *V*_*H*_*1–84* lineage. **a** Multiple sequence alignment of the variable region of heavy chain of mature antibodies with the germline sequence. Mutations in the CDRs and FW regions are highlighted in red. **b** Frequency of *V*_*H*_ mutations and percent identity with corresponding germline sequence. **c** Stereo view of ribbon representation of structure superposition of free antibodies (last structure of simulation) showing the mutated residues of *V*_*H*_ region in sticks. Stereo view of ribbon representation of structure superposition of respective free antibodies (last structure of simulation) and bound forms (crystal structures) of (**d**) Fab ED10 (PDB ID: 2OK0), (**e**) Fab 5.11A1 (PDB ID: 1YJD) and (**f**) anti-uPAR (PDB ID: 3BT2) antibody

Sequence analysis of the variable region of heavy chain in *V*_*H*_*1–84* antibodies suggests that Fab ED10 (as a complex in PDB ID: 2OK0), Fab 5.11A1 (as a complex in PDB ID: 1YJD) and anti-uPAR antibody (as a complex in PDB ID: 3BT2) share 81.25, 83.67 and 85.71% identity with their germline *V*_*H*_*1–84* gene each carrying 17, 16 and 13 *V*_*H*_ mutations respectively in the CDR and framework regions (Fig. 3a and b). Presence of allelic variants of germline gene also contributes to the difference in sequence within a lineage. High-throughput sequencing of human antibody repertoire has reported various polymorphic forms of IGHV and IGHD loci [16, 17], but such a report is rare in mouse [18]. At a few positions, somatic mutation between the antibodies is conserved (V2, D65) while most map to different regions (Fig. 3a, Table 2). A few mutations facilitate interaction with the antigen in Fab 5.11A1 (S31) and anti-uPAR antibody (D55, E58) while most lie in the vicinity of interacting residues (Table 2).

**Table 2:** Somatic mutation and interacting residues in antibodies of *V*_*H*_*5–51* and *V*_*H*_*1–84* lineages

Germline lineage	PDB ID	*V*_*H*_ mutation	Interacting residues on H-chain
*V*_*H*_ *5–51*	4NRX	Q1, V24, A30, E32, K58, I68, N76, I89	S31, W33, D54, D56, Y100C, R100F, T100G
	4HWB	V51, Y58	S32, D56, S57, R60, N102, W34
*V*_*H*_ *1–84*	2OK0	V2, E5, E6, M33, L36, R37, K39, V51, I56, D65, A71, I77, V78, H81, D85, N87,T92	N35, G98
	1YJD	V2, T16, R19, E23, S31, H35, C50, N55,V56, N59, D65, I71, R85, M86, T97	S31,Y101, G102, D104
	3BT2	V2, K3, V11, S28,N31, F32, H35, F52, H52A, D55, E58, D65, A70,	Y33, W50, D55, N56, T57, E58

**Fig. 4:**
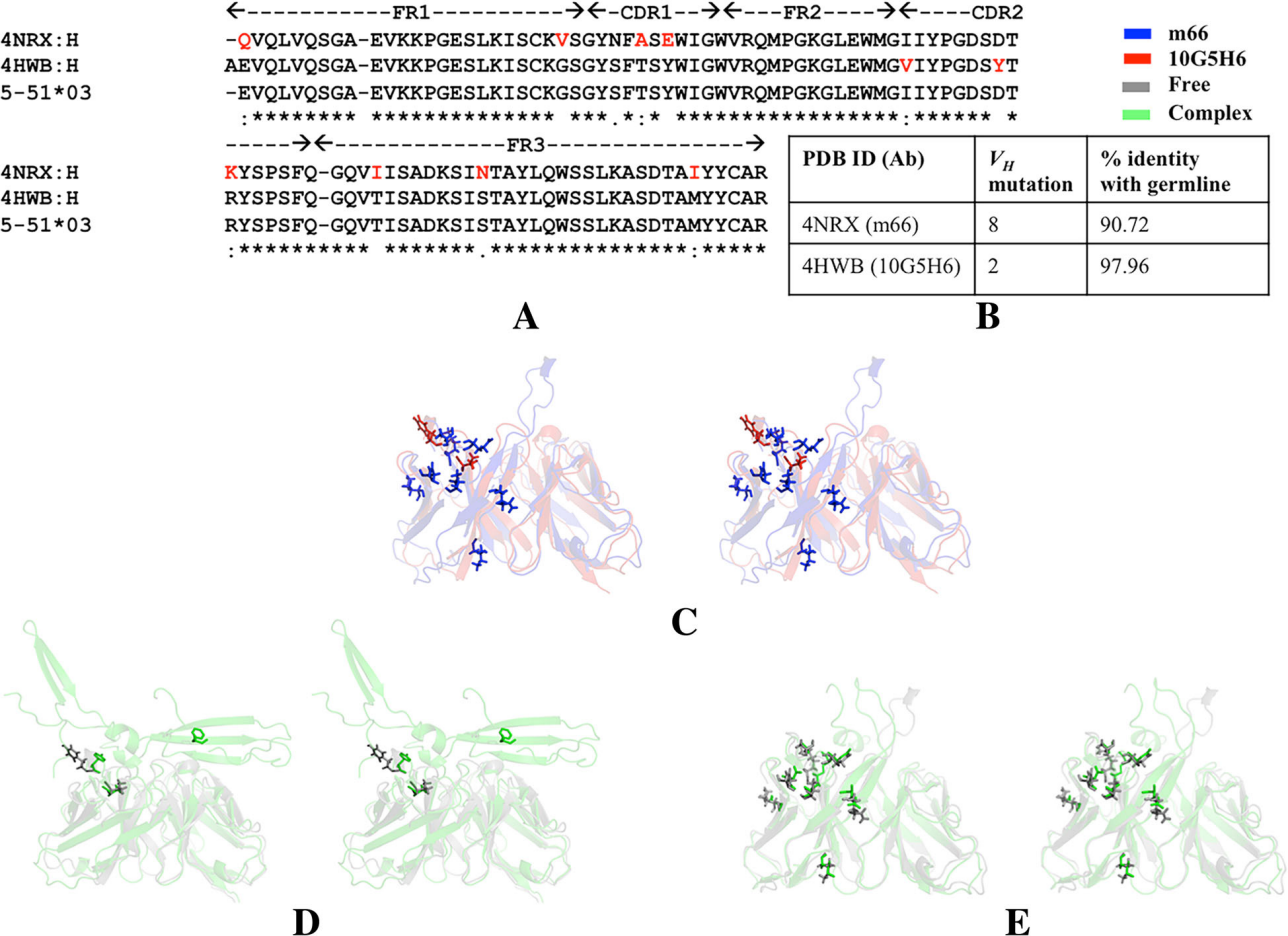
Mapping somatic mutations on sequences and structures of antibodies of *V*_*H*_ *5–51* lineage. **a** Multiple sequence alignment of the variable region of heavy chain of mature antibodies with the germline sequence. Mutations in the CDRs and FW regions are highlighted in red. **b** Frequency of *V*_*H*_ mutations and percent identity with corresponding germline sequence. **c** Stereo view of ribbon representation of structure superposition of free antibodies (last structure of simulation) showing the mutated residues of *V*_*H*_ region in sticks. Stereo view of ribbon representation of structure superposition of respective free antibodies (last structure of simulation) and bound forms (crystal structures) of (**d**) Fab 10G5H6 (PDB ID: 4HWB) and (**e**) Fab m66 (PDB ID: 4NRX)

Sequence analysis of *V*_*H*_*5–51* antibodies suggests that Fab m66 (as a complex in PDB ID: 4NRX) has 90.72% identity with its germline *V*_*H*_*5–51* gene harboring as many as 8 *V*_*H*_ mutations in the CDR and framework regions while Fab 10G5H6 (as a complex in PDB ID: 4HWB) is 97.96% identical carrying 2 *V*_*H*_ mutations (Fig. 4a and b). None of the somatic mutations is involved in interaction with antigen. However, a few of the mutations lie in the vicinity of the interacting residues (Table 2). The number, as well as nature of mutations in both antibodies, is different and they map to different regions (Table 2).

Analysis of multiple sequence alignment of the mature and respective germline forms suggests that mutations are random within a lineage and mutational frequency is high in the CDRs as compared to framework regions. Variations in the framework region contribute to the orientation of *V*_*H*_*-V*_*L*_ pairing and support the antigen-binding site [19, 20]. Despite sequence variation, the canonical structures of CDRs L2 and H1 of *V*_*H*_*1–84* antibodies were conserved as they belonged to class 1 category. CDRL2 of *V*_*H*_*5–51* antibodies assumed a common canonical class 1 category. This indicates that despite somatic mutations led sequence variation, some CDR loops of the antibodies had conserved structural framework. Further examination of the conformational ensemble using molecular dynamics simulation will advance our understanding of the structural principles that govern maturation associated binding to unrelated antigens, while bearing some degree of structural connectivity.

### Conformational selection leads to structural divergence during maturation

Affinity maturation associated changes in *V*_*H*_*1–84* and *V*_*H*_*5–51* lineages were comprehended by carrying out 0.5 μs all-atom molecular dynamics simulation of bound and free mature counterparts. Conformational ensembles were analyzed by subjecting trajectories of bound and respective free forms together to k-mean clustering protocol. Frames with similar conformations could be clustered using Cα radius of 1.5 Å from the centroid. Apart from overall structural variations and connectivity of structural region, changes in the core of the paratope, in particular, were traced.

**Fig. 5:**
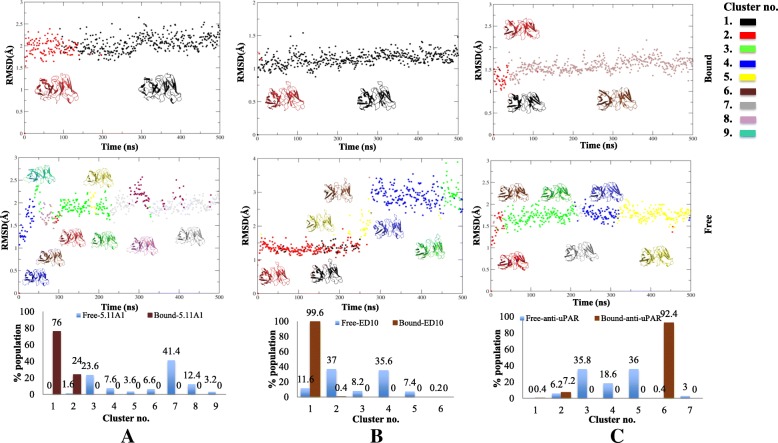
Conformational clusters of *V*_*H*_*1–84* lineage. Sampled conformations were clustered (color coded) and represented in an RMSD versus time plot for respective free and bound (**a**) 5.11A1, (**b**) ED10 and (**c**) anti-uPAR antibodies, across 0.5 μs trajectory. Least RMSD representative antibody of each cluster is shown. Same color code between respective bound and free forms reflects the same cluster. Graphs in the lower panel show percent population of free and bound antibodies in each cluster

**Fig. 6:**
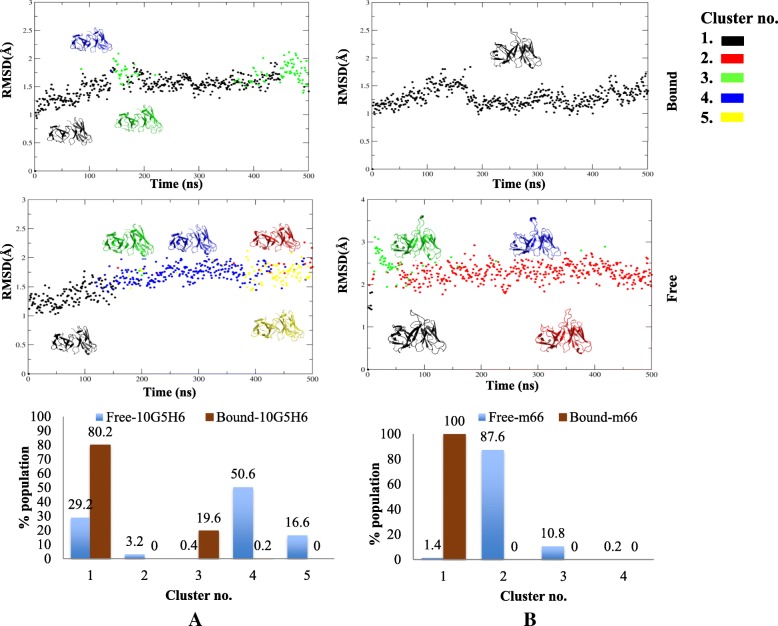
Conformational clusters of *V*_*H*_*5–51* lineage. Sampled conformations were clustered (color coded) and represented in an RMSD versus time plot for respective free and bound (**a**) 10G5H6, (**b**) m66 antibodies across 0.5 μs trajectory. Least RMSD representative antibody of each cluster is shown. Same color code between respective bound and free forms reflects the same cluster. Graphs in the lower panel show percent population of free and bound antibodies in each cluster

Bound antibodies sampled fewer conformers as compared to their free forms. The number of sampled conformers for the free *V*_*H*_*1–84* antibodies was 8 (Fab 5.11A1) and 6 (anti-uPAR, Fab ED10). Bound forms had a highly restricted window of 2 (bound 5.11A1, bound ED10) and 3 (bound uPAR) clusters (Fig. 5). For *V*_*H*_*5–51* lineage, free states sampled 5 (Fab 10G5H6) and 4 (Fab m66) conformers respectively while bound m66 and 10G5H6 Fabs sampled 1 and 2 conformers respectively (Fig. 6). A single molecule was present in one of the clusters of bound 10G5H6, hence it was insignificant to be considered. Thus the alteration in the micro-environment brought about by antigenic stimulus narrows the specificity window of antibody.

Further analysis of the sampled conformers showed that in all cases the bound form of the antibodies selected one from the spectrum of conformers sampled by the free form (same colour code of conformers in Figs. 5 and 6 between bound and free forms represent common cluster). The number of frames in a cluster was expressed as a percentage of the population adopting a conformation (Lower panels in Figs. 5 and 6). Conformation with highest percent population is the dominant conformation of the molecule. In case of ED10 the population of the dominant conformation for DNA bound antibody was 99.6%, while the population of the same conformer was only 11.6% in its free form. For bound anti-uPAR it was 92.4% as against 0.4% in its free form (Lower panel in Figs. 5). Similarly for *V*_*H*_*5–51* antibodies, dominant conformation for bound 10G5H6 was 80.2% as opposed to 29.2% in its free form and the population for bound m66 was 100% as against 1.4% in its free form (Lower panel in Fig. 6). Thus the free form of antibody samples all possible conformers of which one is naturally selected and best optimized for preferential antigen binding. In case of 5.11A1, the free form did not sample the dominant conformation of the bound form. This indicates that the antibody presumably undergoes induced-fit and assumes a different conformation of the paratope to accommodate antigen CD28 (Lower panel in Fig. 5a).

**Fig. 7:**
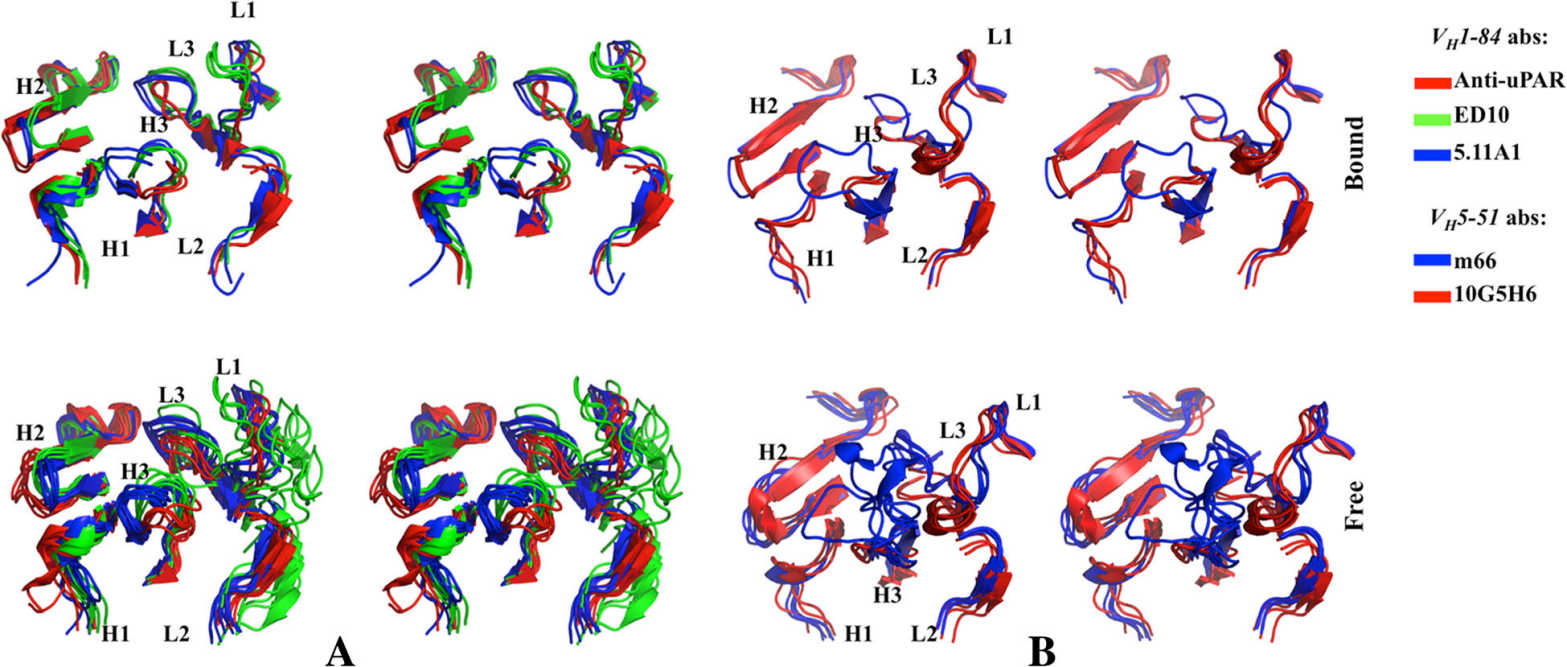
Rigidity in paratope of mature antibodies. Stereo view of ribbon representation of structure superposition of conformers from individual clusters of bound (upper panel) and free (lower panel) antibodies (**a**) anti-uPAR (red), 5.11A1 (blue) and ED10 (green) of *V*_*H*_*1–84* lineage (on the left), and (**b**) 10G5H6 (blue) and m66 (red) of *V*_*H*_*5–51* lineage (on the right)

Representative frames from conformational landscape of bound as well as free forms were analyzed by structure superposition to assess changes in the paratope. Each of the bound mature variants adopted a distinct conformation that did not overlap conformational window of the other bound antibodies. The landscape remained highly restricted; corresponding to the native crystal structures (upper panel in Fig. 7). This was true for the free forms as well barring the long loops of CDRL1 in ED10 which is also reflected as jumps in the RMSD plot (Fig. 5b) and CDRH3 in m66 that showed relatively wide conformational space (lower panel in Fig. 7). As observed in the compiled data, variability of CDRs of both chains was apparent within a lineage across the dataset. So, we evaluated the dynamics of the loops in the complexes of both lineages. While variability was seen in all loops, CDRH2 was relatively less impacted by flexibility of these loops. The mobility of CDRH3 was complemented by the relative shifting of VL and marginally by CDRH1 in *V*_*H*_*1–84* lineage. Apparently, since the conformational spectrum was narrow and the conformers of individual antibody showed good alignment in both lineages, not much information could be drawn. While the paratope in each case seemed to have diversified, pairwise comparison of all conformers and crystal structures suggested very similar topology of the backbone between sibling antibodies. Additional file 3: Figures S5 and S6 show heatmaps generated from RMSD matrix obtained from a one-to-one comparison of all conformers and crystal structures (that included the entire variable regions composed of CDRs and framework regions). Blue regions indicate conformational integrity between the antibodies presumably reflecting structural imprint of their common lineage. These findings suggest that despite an overall backbone structural connectivity, conformational selection of the paratope is imperative to favour preferential antigen binding within the available space. Our results are in conformity with the canonical theory of affinity maturation that suggests an optimized binding pocket while following isolated molecular pathways of maturation [13, 21].

### Structural dynamics in light of *V*_*H*_ mutation

In order to check if the structural divergence between the mature antibodies is due to somatic mutation or the degree of variability increases due to binding of incoming antigen, the last structure of free antibody obtained from simulation was compared with that of the crystal structure of bound antibody by structure superposition. Superposition of the free states of all antibodies obtained from simulation suggested structural divergence (Figs. 3c and 4c). Pairwise structural alignment of the free and respective bound states showed significant variation in the paratope region and particularly in mutated residues (Figs. 3d-f and 4d-e). Thus it can be said that while mutations tailor the overall geometry of individual antibodies of a lineage, it is the interaction with respective antigen that leads to additional topological alteration in their paratope.

### Analyses of bonding pattern and binding energy of the complexes

The trajectories were further examined to check interface bonding pattern. For distance cut off of 3.5 Å, H-bonds between antibody and antigen were calculated using CPPTRAJ module of AMBER14. H-bonds with or above 30% occupancy across the trajectories are presented in Additional file 3: Tables S3 and S4. In *V*_*H*_*1–84* lineage, anti-uPAR complex had 15 bonds (ASN_190 and TRP_325, GLY_217 and TYR_308, GLN_189 and TYR_483 being the most stable ones with 93, 93 and 88% occupancy respectively), Fab 5.11A1 complex had 4 (stable ones being GLU_316 and TYR_205, GLY_206 and TYR_280 each with 49% occupancy) and Fab ED10 complex had 6 H-bonds (with the highest occupancy being 66% between different atoms of DT5_1 with ASN_147 and TYR_145) (Additional file 3: Table S3). Fab m66 and Fab 10G5H6 complexes of *V*_*H*_*5–51* lineage had 10 (stable being between TYR_107 and SER_245 with 74% occupancy and between LEU_240 and TRP_33 with 73% occupancy) and 15 H-bonds (most stable was between TYR_318 and LYS_93 with 94% occupancy) respectively (Additional file 3: Table S4). Different sets of H-bonds in the trajectories of the mature variants was a manifestation of mutations during maturation, suggesting independent developmental pathways. Binding surfaces for individual antigens followed the standard principle [22]. A flattened surface was assumed by anti-protein antibodies viz. anti-uPAR, Fab 5.11A1 and Fab 10G5H6 antibodies as opposed to anti-peptide antibody (Fab m66). Due to the small size of DNA fragment, Fab ED10 showed a small groovy binding site buried deeper in the *V*_*H*_*-V*_*L*_ interface. Together these findings suggest how the physicochemical factors govern reorganization to foster shape complementarity.

**Fig. 8:**
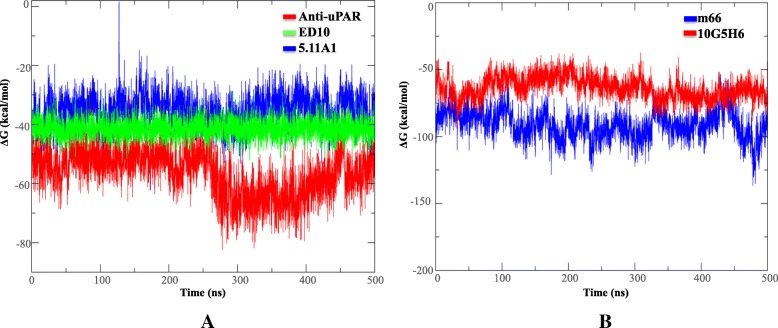
Binding energy of antibodies for respective antigens. ΔG values obtained from MM-GB/SA analysis have been plotted across the trajectories of (**a**) 5.11A1 (blue), anti-uPAR (red) and ED10 (green) from *V*_*H*_*1–84* lineage and (**b**) m66 (blue) and 10G5H6 (red) from *V*_*H*_*5–51* lineage

Change in overall number of H-bonds during maturation has been linked with the type of antigen bound, but, its direct association with binding energetics has not been established [23–26]. As a measure of affinity, the free energy of binding (ΔG) was calculated from the trajectories using molecular mechanics generalized Born surface area (MM-GB/SA) approach. While entire 0.5 μs trajectories were used for computing binding free energies, average ΔG for each complex was evaluated from the last 100 ns of the trajectories. The average ΔG was − 38.75 kcal/mol for Fab 5.11A1 complex, − 53.38 kcal/mol for anti-uPAR complex and − 42.45 kcal/mol for Fab ED10 complex of *V*_*H*_*1–84* lineage (Fig. 8a). In *V*_*H*_*5–51* lineage, average ΔG was − 92.48 kcal/mol for Fab m66 complex and − 68.28 kcal/mol for Fab 10G5H6 complex (Fig. 8b). Based on past studies, relative free binding energies were calculated by excluding entropy from the analysis as it is not considered to be predictive of accuracy [27, 28]. Hence, the values obtained for the antibody complexes are not exact match but relative values between different states that correlate with experimental values. The rule identified for governing affinity between complexes is largely dictated by the nature of ligand /antigen. In protein-protein systems studied till date, it is seen that affinity maturation contribute modestly to the binding energetics as compared to protein-hapten systems [25, 26]. For haptens, the increase in affinity is attributable to additional H-bonds, co-operative binding or significant structural alterations while the same is not true for proteins [29]. Different sets of attractive interactions affect the binding energies by an increase in enthalpic gain [11].

## Discussion

The study was aimed at understanding what factors shape antibodies coming from a common germline lineage to be able to bind significantly distinct antigens. While there are past reports that have analyzed maturation associated evolution, the contexts, however, were different from ours. Some reports are based on sequence analyses alone while some emphasize on dynamics of antibody evolution to a common epitope. The prime focus of our study was to strictly examine structural diversification of mature antibodies for a wide range of chemically distinct antigens. The approach of the study is data-driven, where available crystallographic structures have been analyzed and inferences drawn are validated by characterizing dynamics within lineages.

Analyses of static structural data from 35 (mouse) and 13 (human) IGHV families coupled with different light chain genes revealed that antibodies of a certain descent undergo topological tailoring of their binding pockets mediated by all the CDR loops to accommodate distinct antigens. The contribution of individual loop, however, varies across lineages. The dataset transcends a wide range of antigens covering hapten (24% in mouse and 5% in human), peptide (27% in mouse and 21% in human), sugar (5% in mouse and 5% in human), protein (41% in mouse and 69% in human) and nucleic acid (3% in mouse) (Fig. 1) that are typical in pathogens. Hence, conceptually our study is of credence as the diversity of epitopes facilitates a realistic understanding of host-pathogen interaction.

Sequence examination of two lineages indicates that different number and nature of mutations differentiate the antibodies from the common germline precursor. During affinity maturation, repeated rounds of mutations result in various intermediate stages of sequence diversification; hence the antibodies presumably represent different stages of evolution each following an isolated maturation pathway [30]. If the extent of maturation of the reported structures could be investigated, additional insights could have been drawn. Sequence variations within the hypervariable regions shift the canonical structure framework relative to individual CDR loops to accommodate distinct antigens and maintain complementarity of interacting surfaces by reducing entropic cost [11, 31]. Further conformational landscape reveals that despite sequence variation led structural re-arrangement, the overall backbone geometry is similar. It can be envisaged that a common structural imprint of the germline is inherited. This suggests that while affinity maturation is highly stochastic, the evolution of the antibody repertoire is shaped by structural constraints. Examination of paratope sheds light on how one conformational state is naturally selected from the available spectrum and optimized to favor antigen binding. It is the optimization that brings about divergence in the paratope of individual antibody; evident from non-overlapping paratope topologies. Studies that reported maturation associated pre-ordering of the antibody combining site to favor antigen binding corroborates these findings [24, 32–35]. Present concepts on protein structure and function postulate coexistence of several functional states even for a highly specific enzyme. It is the population of a distinct conformation that determines the specificity of the molecule [36]. The observed homogeneity in bound antibody indicates selection of a functional state, thereby demonstrating narrowing down of specificity window leading to molecular divergence during affinity maturation [35, 37–39]. An analogy to this can be quoted in Darwin’s words from The Origin of Species, “from so simple a beginning endless forms most beautiful and most wonderful have been, and are being, evolved” [40].

It may be noted that the observations made from simulation studies provide an interesting perspective to the interrogation of structural changes in sibling antibodies that target different antigens. Whether the antigen is self or foreign has no bearing on our observation as the study primarily dwells with identifying structural principles of interaction governing evolution. Due to practical limitations, conclusions have been drawn from simulations of 5 complexes from 2 IGHV lineages. Subjecting more complexes to simulation would increase the confidence of the interpretation. Additionally, the hypothesis derived from the study, as is the case with all computational analyses, has to be further validated experimentally. The best experimental follow-up of this study would be to make recombinant germline antibodies and determine their three dimensional structures by X-ray diffraction in antigen bound and unbound states. Subsequently, simulation studies can be conducted to sample conformational space of the germline complexes and compare them with the mature counterparts to decipher structural diversification in the lineage.

## Conclusion

The computational analysis of global data followed by dynamics illustrates the molecular mechanism of how modulation in the structure is paramount to biomolecular recognition. Data from various computational approaches used in this study support the existence of different routes of maturation. Structural adaptation of the paratope despite conservation of overall backbone architecture is a pivotal finding of the study. The results of the study present an interesting implication of molecular evolution leading to the generation of antibody diversity. Additionally, our analysis displays characteristic that can perpetuate the antibody diversity model [41] whereby the 4th level of diversity is attained after somatic diversification of a lineage leading to recognition of diverse antigens.

## Methods

### Data retrieval and compilation

Coordinate files of Ab-Ag complexes were retrieved from RCSB PDB (www.rcsb.org/pdb). Data so obtained were filtered and subjected to mining as mentioned in the result section. CDRs were identified using Kabat numbering system [42]. Coordinate files of complexes were retrieved and segregated based on source of antibody into two groups, human and mouse. While more than 90% of the retrieved antibodies were obtained from immunized house mouse, the strain of the mouse was not considered as a criterion for selection of the antibodies as no two individuals have identical genetic make up, hence the immune repertoire would also vary.

### Identification of germline origin of the antibodies and their clustering

Candidate sequences were queried for germline genes in IMGT Database using Ig BLAST tool 1.3.0 at NCBI with default settings [43, 44]. The antibodies were then clustered based on common germline *V*_*H*_ origin. Data sharing no common origin, were discarded while the rest were grouped.

### Structural analysis

Structure based sequence alignment of all the lineages were performed in Chimera 1.11.2 [45]. One antibody was randomly chosen as reference with which other structures were matched. RMSDs of CDRs were noted. Contacts between the complexes were noted from PDBsum [46]. For multi-subunit antigen, the bonds between antibody and each chain of the antigen are added and reported. In complexes where PDBsum did not fetch any interaction information, PISA 1.48 [47] was used to identify the contacts.

### Selection of system

Three mature antibodies, anti-uPAR, Fab 5.11A1 and Fab ED10 that bound to uPAR, CD28 and DNA respectively of *V*_*H*_*1–84* lineage from mouse formed a system (PDB ID: 3BT2, 1YJD, 2OK0 respectively). Two of four antibodies, Fab 10G5H6 bound with ectodomain D3 of IL-13 and antibody m66 bound with gp41 MPER (Membrane Proximal External Region) peptide (PDB ID: 4HWB, 4NRX respectively) of *V*_*H*_*5–51* lineage from human comprised of the other system and were chosen for simulation. Since in human, the other 2 antibodies Fab 2558 and Fab CH58 also bound to peptides, therefore these were excluded in the study to maintain distinctness of epitope. Only molecules directly interacting with the antibody were retained, rest were deleted. For antibody, only Fv (fragment variable) region constituting of CDRs and framework regions was retained. Antigens from each of the complexes were removed to generate free form of antibodies.

### Sequence analysis

Multiple sequence analysis of the antibodies and their corresponding germline V_*H*_-gene was performed online using CLUSTAL omega (default settings) because of accuracy of alignment [48]. Mutations were identified from the alignment. Percent identity matrix was generated from the alignment to obtain identity between the sequence with germline counterpart. Canonical classes of the CDRs were assigned using strict Chothia SDR templates (http://www.bioinf.org.uk/abs/chothia.html) [12, 31].

### Molecular dynamics simulation

#### Simulation set-up

All the starting heteromeric structures were provided as input to tLEaP module in AMBER14 package to generate topology and coordinate files [49]. Molecular mechanics parameters were assigned using ff12SB force-field [50]. The molecules were explicitly solvated using TIP3P water box with box edges lying 10 Å from the outermost atoms of the proteins in all directions. Charge of the system was neutralized with monovalent counter ions, Na + or Cl-. Prior to subjecting to simulation, energy minimization was performed for 5000 steps with steepest descent for first 2500 steps followed by conjugate gradient for rest. If steric clashes persisted, minimization cycle was increased. Systems were heated to 300 K during a 14 ps dynamics simulation using the NVT ensemble. The temperature of the system was constrained using Langevin dynamics temperature coupling with a time step of 2 fs. Pressure was equilibrated to 1 atm over a period of 10 ps using isotropic position scaling keeping the temperature constant at 300 K. A third equilibration was run for 100 ps to stabilize the system. Production MD run was conducted using the NPT ensemble for 0.5 μs at 300 K and 1 atm for each system. Snapshots were saved at an interval of 10 ps. All the MD simulations were performed using Sander and a parallel CUDA version of PMEMD from AMBER14 [51, 52]. All simulations were performed in-house using High Performance Computing (HPC) facility with NVIDIA K20X GPUs.

#### Analysis of MD trajectories

MD trajectories were visualized in Visual Molecular Dynamics (VMD) [53] and analyzed using CPPTRAJ module of AMBER14 [54]. Structure superpositions of the frames were done using PyMOL 1.8.0.6. To analyze the extent of conformational sampling and to compare conformers obtained, snapshots extracted at an interval of 1 ns from the 0.5 μs trajectories were clustered together. Clustering was implemented between respective free and bound forms by subjecting to k-mean clustering with 1.5 Å radius from the centroid using the kclust utility in MMTSB (Multiscale Modeling Tools for Structural Biology) suite [55]. The k-means clustering tries to find k groups from n data points depending on their nearest mean to each cluster [56]. Conformational landscape obtained from clustering are depicted in an RMSD vs time plot where different colors correspond to different clusters. Highly flexible residues far from the binding pocket (if any) were removed during RMSD calculation. To decipher overall structural correlation between all the conformers, their backbone atoms were aligned and a heatmap was generated with the pairwise RMSD scores using pheatmap package of R [57]. Somatic mutations identified from sequence analysis were mapped in the frames. For contact assignment, H-bond interactions were noted. Side-chain hydrogen bond distance cut-off was set to 3.5 Å between donor/acceptor atoms for frames extracted at 100 ps. Molecular mechanics generalized Born surface area (MM-GB/SA) was used for a relative comparison of free energy of interaction between the complexes [58]. The method was implemented using MMPBSA.py utilizing generalized Born (GB) implicit-solvent model [58]. Free energy of binding was calculated according to the following equation:

$$ {\Delta \mathrm{G}}_{\mathrm{bind}}\kern0.5em ={\Delta \mathrm{G}}_{\mathrm{complex}}\kern0.5em -{\Delta \mathrm{G}}_{\mathrm{receptor}}\kern0.5em -{\Delta \mathrm{G}}_{\mathrm{ligand}} $$

## Additional files

Additional file 1: **Table S1.** Mouse antibodies of common germline VH (variable region of heavy chain) lineages complexed with antigens. PDB IDs of complexes tabulated. (XLSX 34 kb)

Additional file 2: **Table S2.** Human antibodies of common germline VH (variable region of heavy chain) lineages complexed with antigens. PDB IDs of complexes tabulated. (XLSX 30 kb)

Additional file 3: **Figure S1.** Conformational heterogeneity in mouse data. Stereo images of structure superposition of mouse antibodies of a lineage showing variability in CDRs. **Figure S2.** Conformational heterogeneity in human data. Stereo images of structure superposition of human antibodies of a lineage showing variability in CDRs. **Figure S3.** Contact analysis in mouse data. Stacked bar diagram representing number of H-bonds formed by CDRs of H and L chains of mouse antibody with bound antigens. PDB IDs of antibody complexes belonging to germline VH lineages are represented along Y-axis and number of H-bonds formed by each CDR loop is represented along X-axis. **Figure S4.** Contact analysis in human data. Stacked bar diagram representing number of H-bonds formed by CDRs of H and L chains of human antibody with bound antigens. PDB IDs of antibody complexes belonging to germline VH lineages are represented along Y-axis and number of H-bonds formed by each CDR loop is represented along X-axis. **Figure S5.** Heatmap of RMSD between conformers and crystal structures of antibodies of *V*_*H*_*1-84* origin. Pair wise structural comparison of crystal structures and all conformers (bound and free) obtained after clustering of antibodies of *V*_*H*_*1-84* origin from mouse, plotted along X-axis and Y-axis. Names of all conformers end with a number to represent the clusters. Crystal structures are named as bound-5.11A1 (PDB ID: 1YJD), bound-ED10 (PDB ID: 2OK0) and bound-anti-uPAR (PDB ID: 3BT2). Result is shown as a measure of RMSD in a gradient from blue (low) to red (high). **Figure S6.** Heatmap of RMSD between conformers and crystal structures of antibodies of *V*_*H*_*5-51* origin. Pair wise structural comparison of crystal structures and all conformers (bound and free) obtained after clustering of antibodies of *V*_*H*_*5-51* origin from human, plotted along X-axis and Y-axis. Names of all conformers end with a number to represent the clusters. Crystal structures are named as bound-m66 (PDB ID: 4NRX) and bound-10G5H6 (PDB ID: 4HWB). Result is shown as a measure of RMSD in a gradient from blue (low) to red (high). **Table S3.** H-bond (above 30 % occupancy across trajectory) of antibody complexes of mouse *V*_*H*_*1-84* lineage. **Table S4.** H-bond (above 30 % occupancy across trajectory) of antibody complexes of human *V*_*H*_*5-51* lineage. (PDF 8090 kb)

## Abbreviations

Ab: antibody
Ab-Ag: antibody-antigen
Ag: antigen
CDR: Complementarity Determining Region
Gp: glyco-protein
IL: Interleukin
MD: Molecular dynamics
MPER: Membrane Proximal External Region
PDB: Protein Data Bank
PISA: Proteins, Interfaces, Structures and Assemblies
RMSD: Root Mean Square Deviation
VDJ: Variable Diversity Junction
*V*_*H*_: Variable region of heavy chain
*V*_*L*_: Variable region of light chain
