# Supplementary material for: Deciphering evolution of immune recognition in antibodies
Source: BMC Struct Biol. 2018 Dec 19;18:19. doi: 10.1186/s12900-018-0096-1 (PMC6299584; doi:10.1186/s12900-018-0096-1)
Supplement: Supplementary file 3 — Figure S1. Conformational heterogeneity in mouse data. Stereo images of structure superposition of mouse antibodies of a lineage showing variability in CDRs. Figure S2. Conformational heterogeneity in human data. Stereo images of structure superposition of human antibodies of a lineage showing variability in CDRs. Figure S3. Contact analysis in mouse data. Stacked bar diagram representing number of H-bonds formed by CDRs of H and L chains of mouse antibody with bound antigens. PDB IDs of antibody complexes belonging to germline VH lineages are represented along Y-axis and number of H-bonds formed by each CDR loop is represented along X-axis. Figure S4. Contact analysis in human data. Stacked bar diagram representing number of H-bonds formed by CDRs of H and L chains of human antibody with bound antigens. PDB IDs of antibody complexes belonging to germline VH lineages are represented along Y-axis and number of H-bonds formed by each CDR loop is represented along X-axis. Figure S5. Heatmap of RMSD between conformers and crystal structures of antibodies of VH1-84 origin. Pair wise structural comparison of crystal structures and all conformers (bound and free) obtained after clustering of antibodies of VH1-84 origin from mouse, plotted along X-axis and Y-axis. Names of all conformers end with a number to represent the clusters. Crystal structures are named as bound-5.11A1 (PDB ID: 1YJD), bound-ED10 (PDB ID: 2OK0) and bound-anti-uPAR (PDB ID: 3BT2). Result is shown as a measure of RMSD in a gradient from blue (low) to red (high). Figure S6. Heatmap of RMSD between conformers and crystal structures of antibodies of VH5-51 origin. Pair wise structural comparison of crystal structures and all conformers (bound and free) obtained after clustering of antibodies of VH5-51 origin from human, plotted along X-axis and Y-axis. Names of all conformers end with a number to represent the clusters. Crystal structures are named as bound-m66 (PDB ID: 4NRX) and bound-10G5H6 (PDB ID: 4HWB [file 12900_2018_96_MOESM3_ESM.pdf]

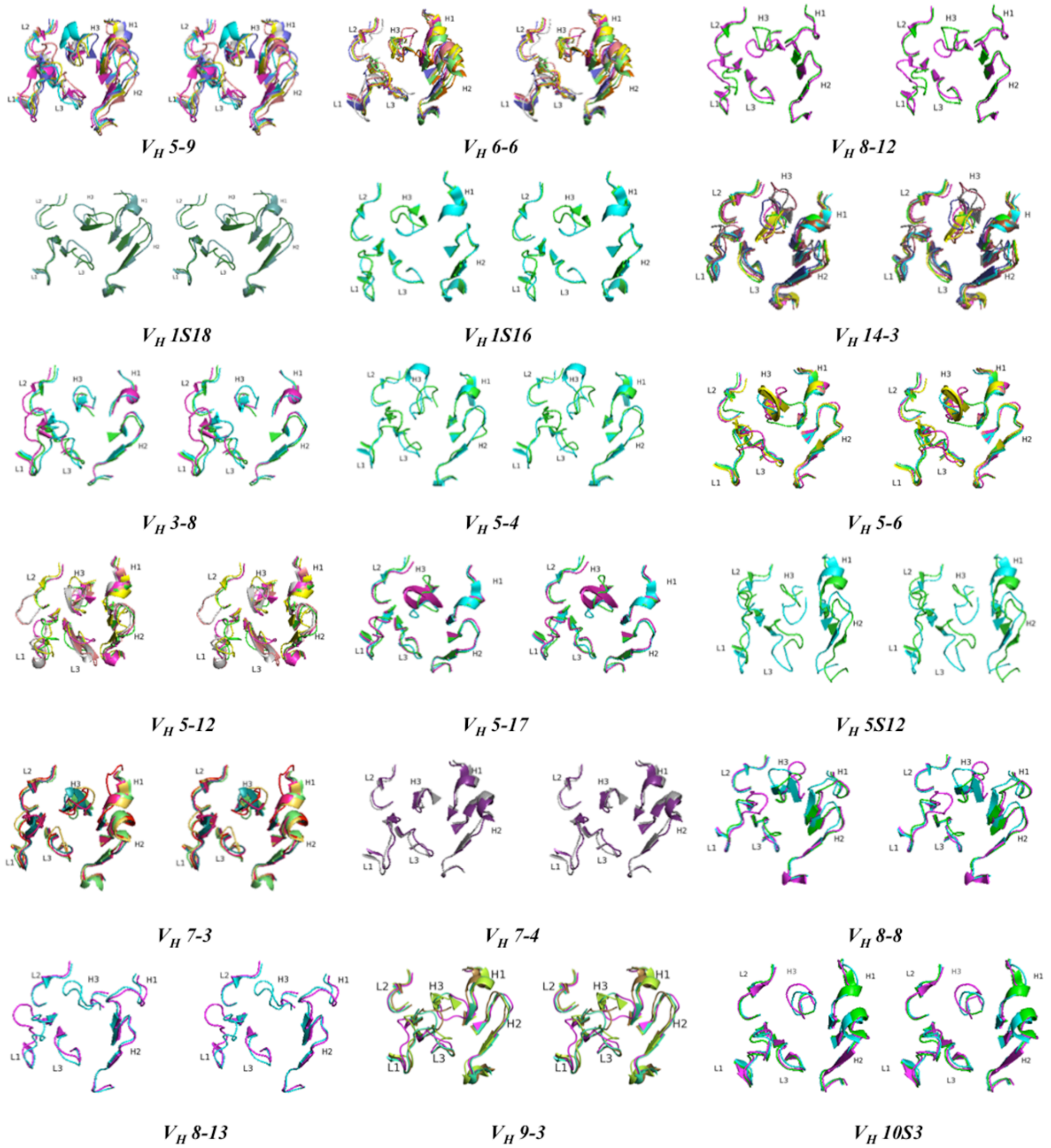

Figure S1. Conformational heterogeneity in mouse data. Stereo images of structure superposition of mouse antibodies of a lineage showing variability in CDRs.

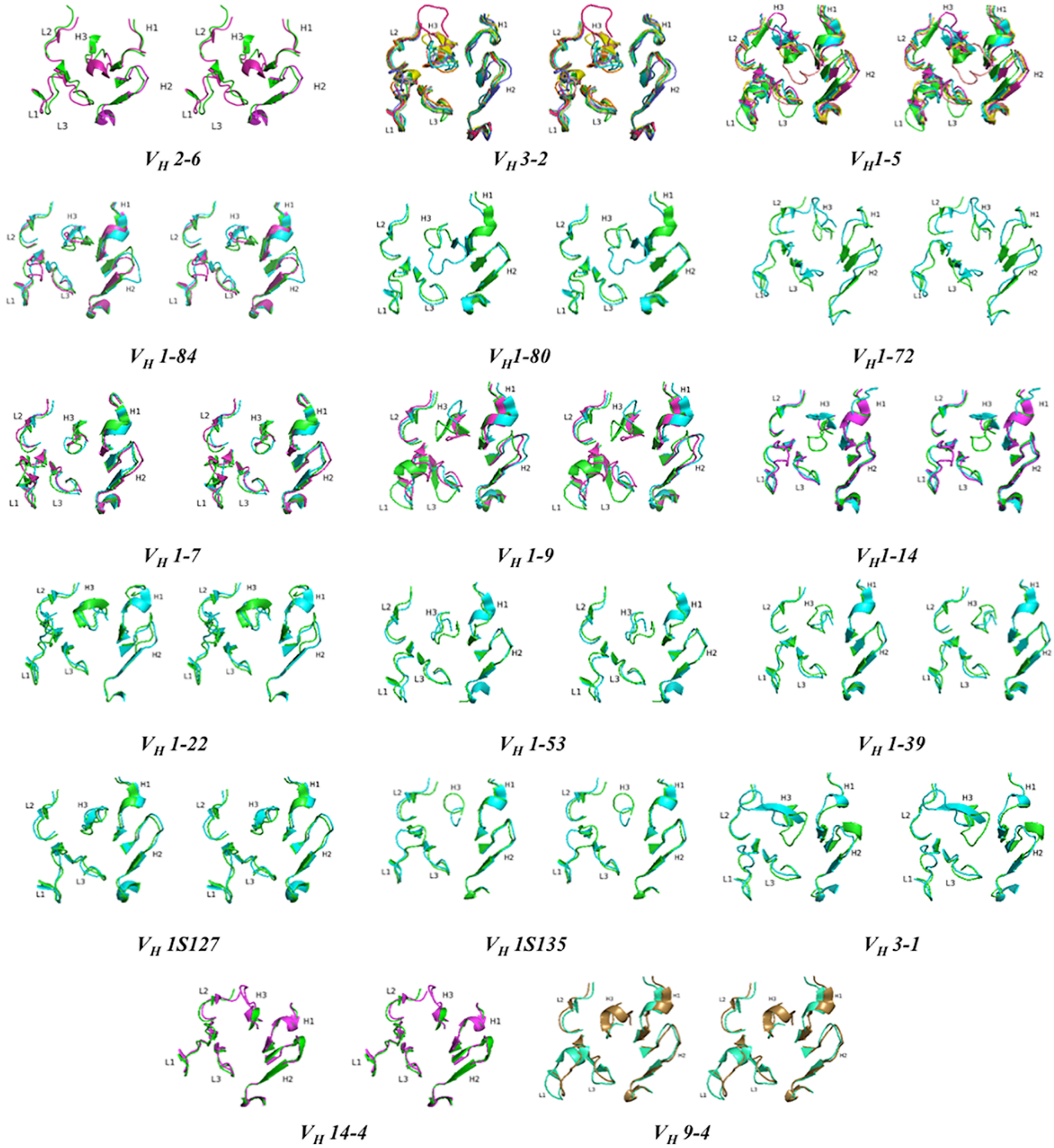

Figure S1 (cont.)

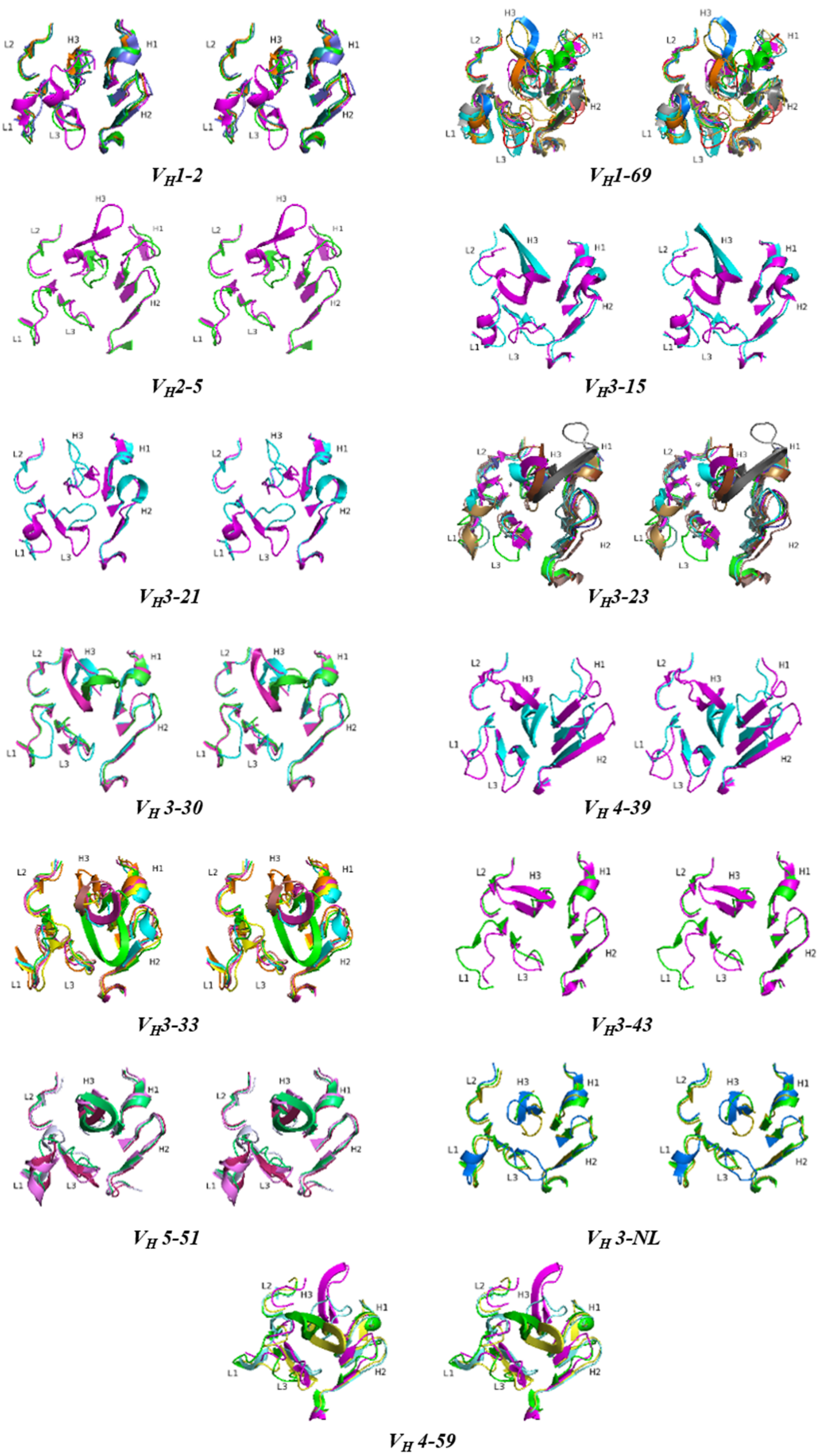

Figure S2. Conformational heterogeneity in human data. Stereo images of structure superposition of human antibodies of a lineage showing variability in CDRs.

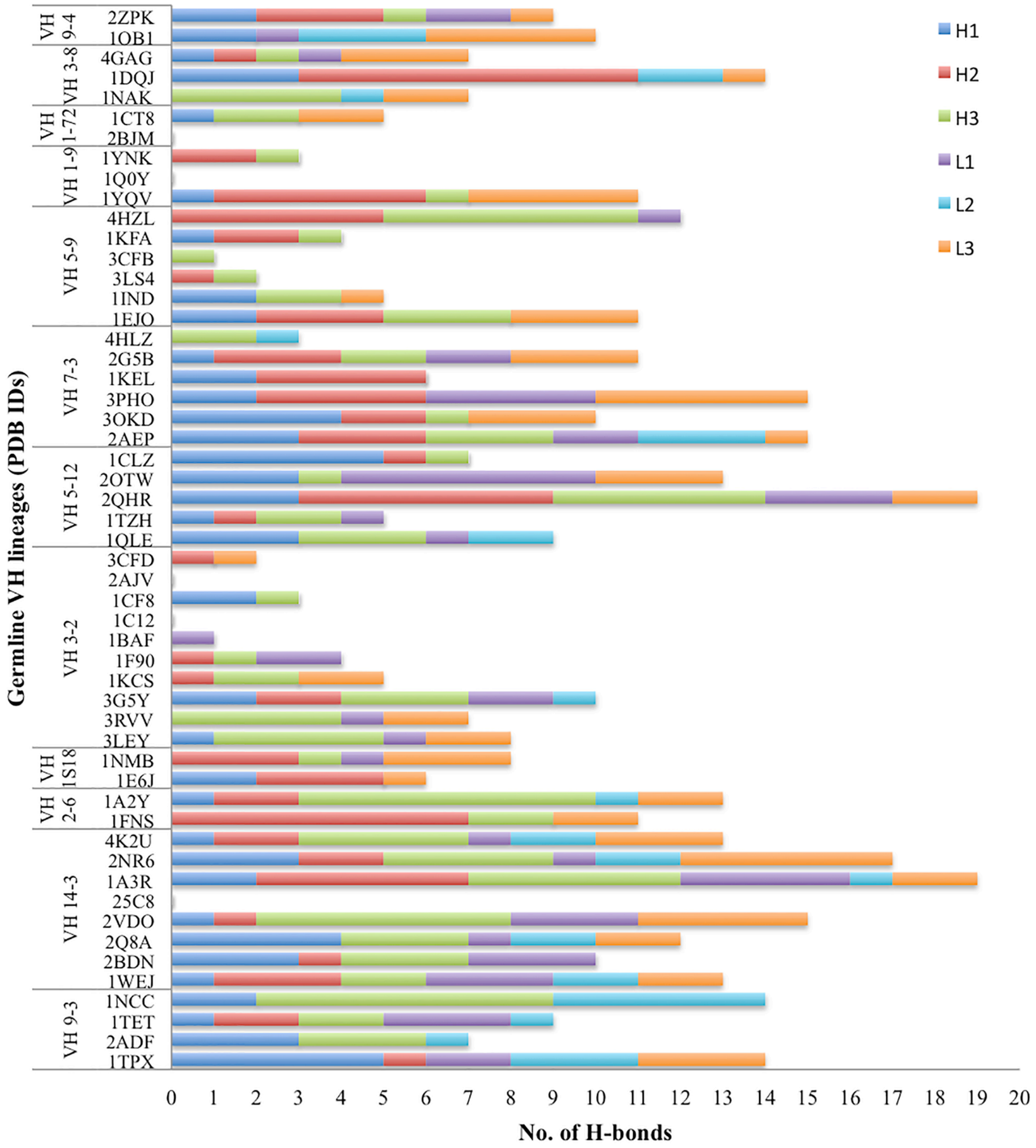

Figure S3. Contact analysis in mouse data. Stacked bar diagram representing number of H-bonds formed by CDRs of H and L chains of mouse antibody with bound antigens. PDB IDs of antibody complexes belonging to germline VH lineages are represented along Y-axis and number of H-bonds formed by each CDR loop is represented along X-axis.

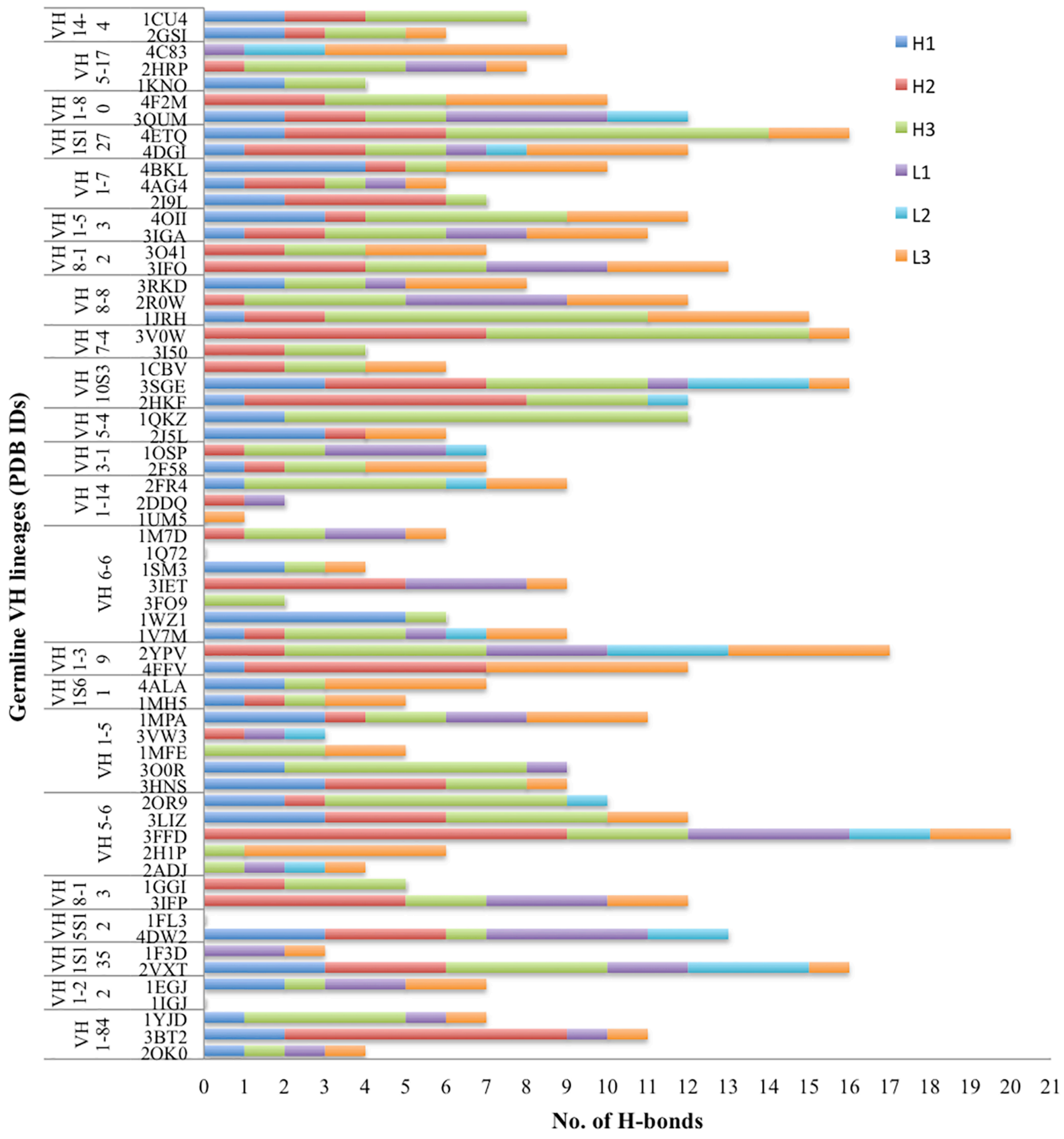

Figure S3 (cont.)

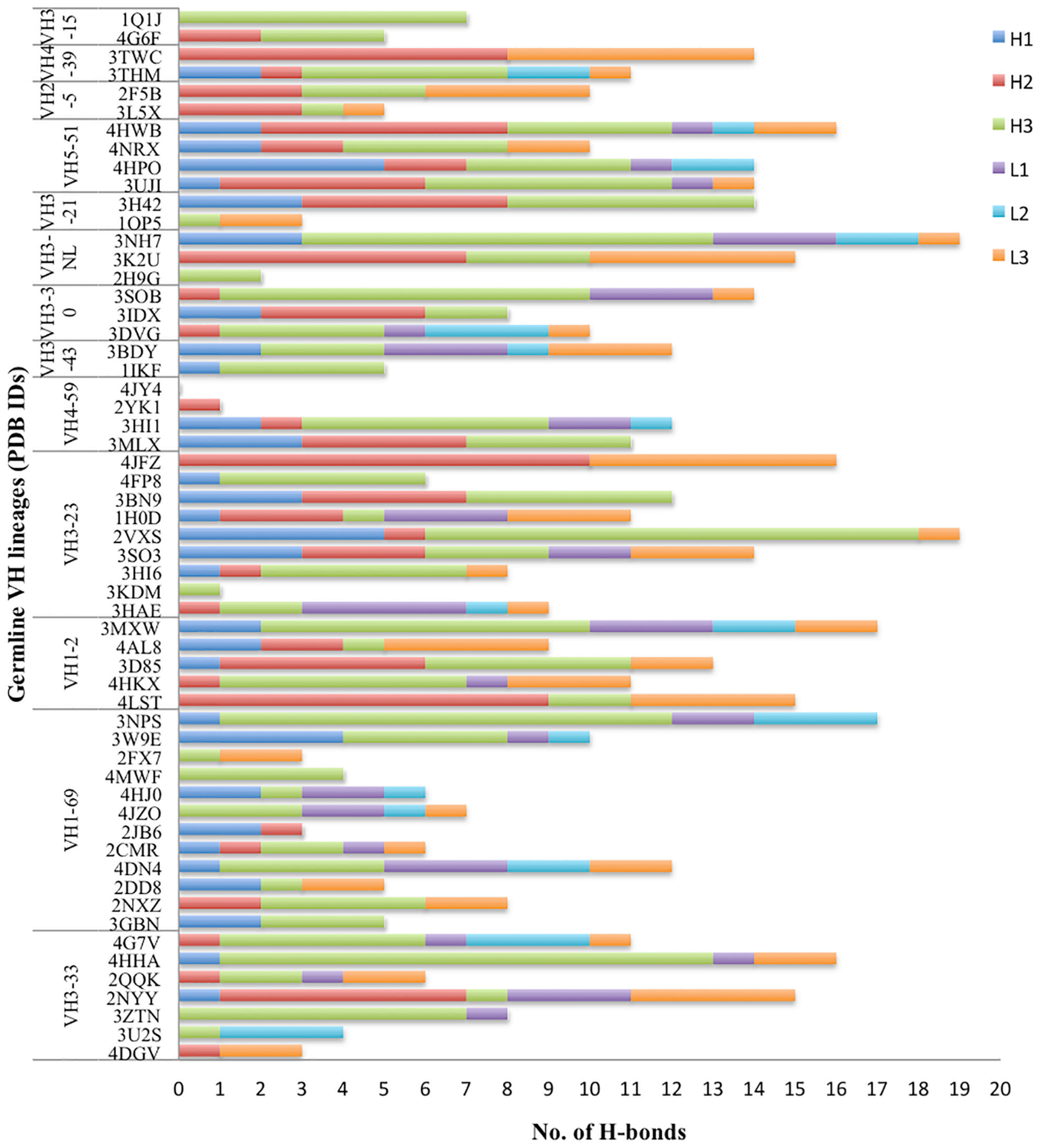

Figure S4. Contact analysis in human data. Stacked bar diagram representing number of H-bonds formed by CDRs of H and L chain of human antibody with bound antigens. PDB IDs of antibody complexes belonging to germline VH lineages are represented along Y-axis and number of H-bonds formed by each CDR loop is represented along X-axis.

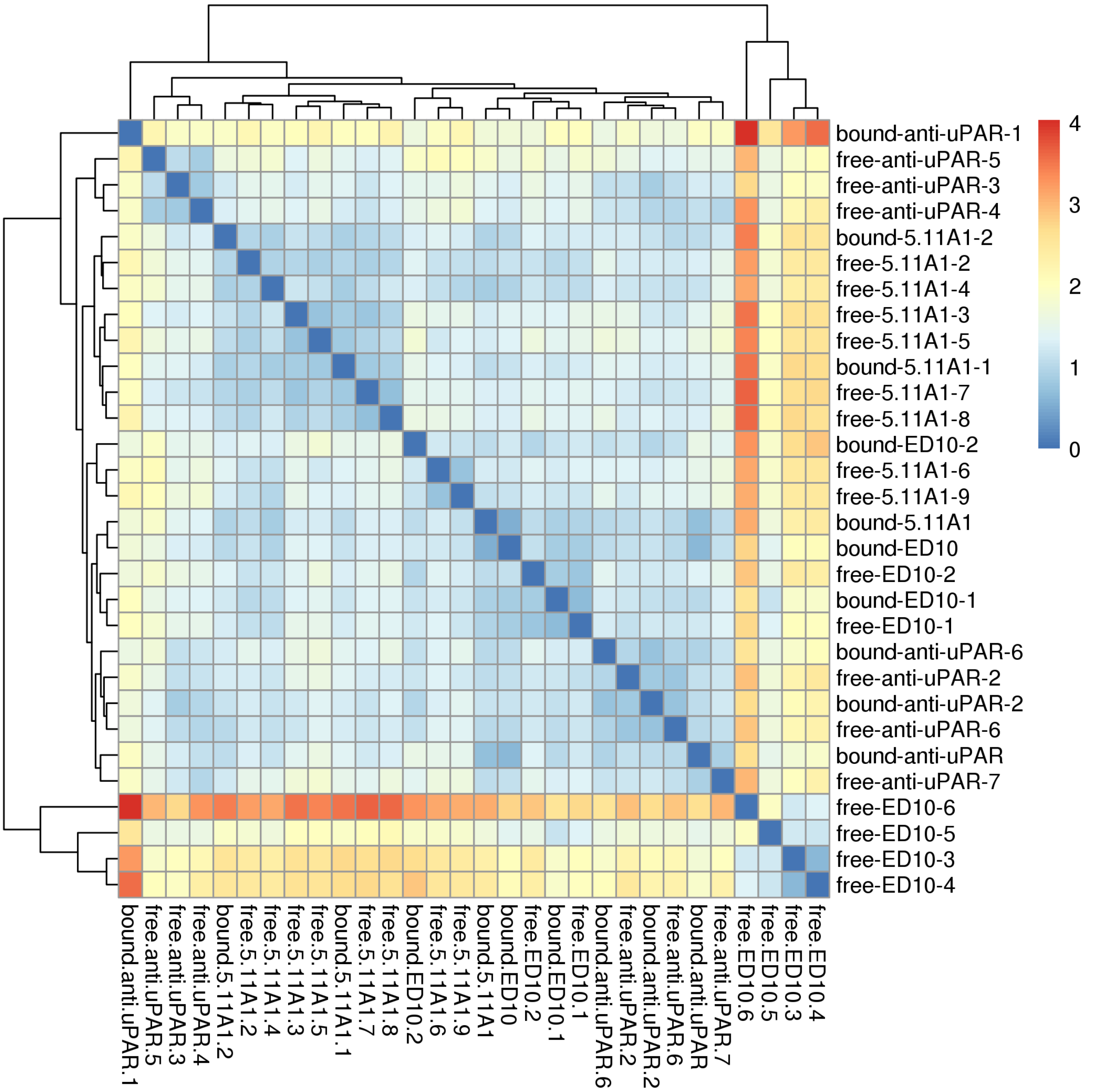

Figure S5. Heatmap of RMSD between conformers and crystal structures of antibodies of *V_H1-84* origin. Pair wise structural comparison of crystal structures and all conformers (bound and free) obtained after clustering of antibodies of *V_H1-84* origin from mouse, plotted along X-axis and Y-axis. Names of all conformers end with a number to represent the clusters. Crystal structures are named as bound-5.11A1 (PDB ID: 1YJD), bound-ED10 (PDB ID: 2OK0) and bound-anti-uPAR (PDB ID: 3BT2). Result is shown as a measure of RMSD in a gradient from blue (low) to red (high).

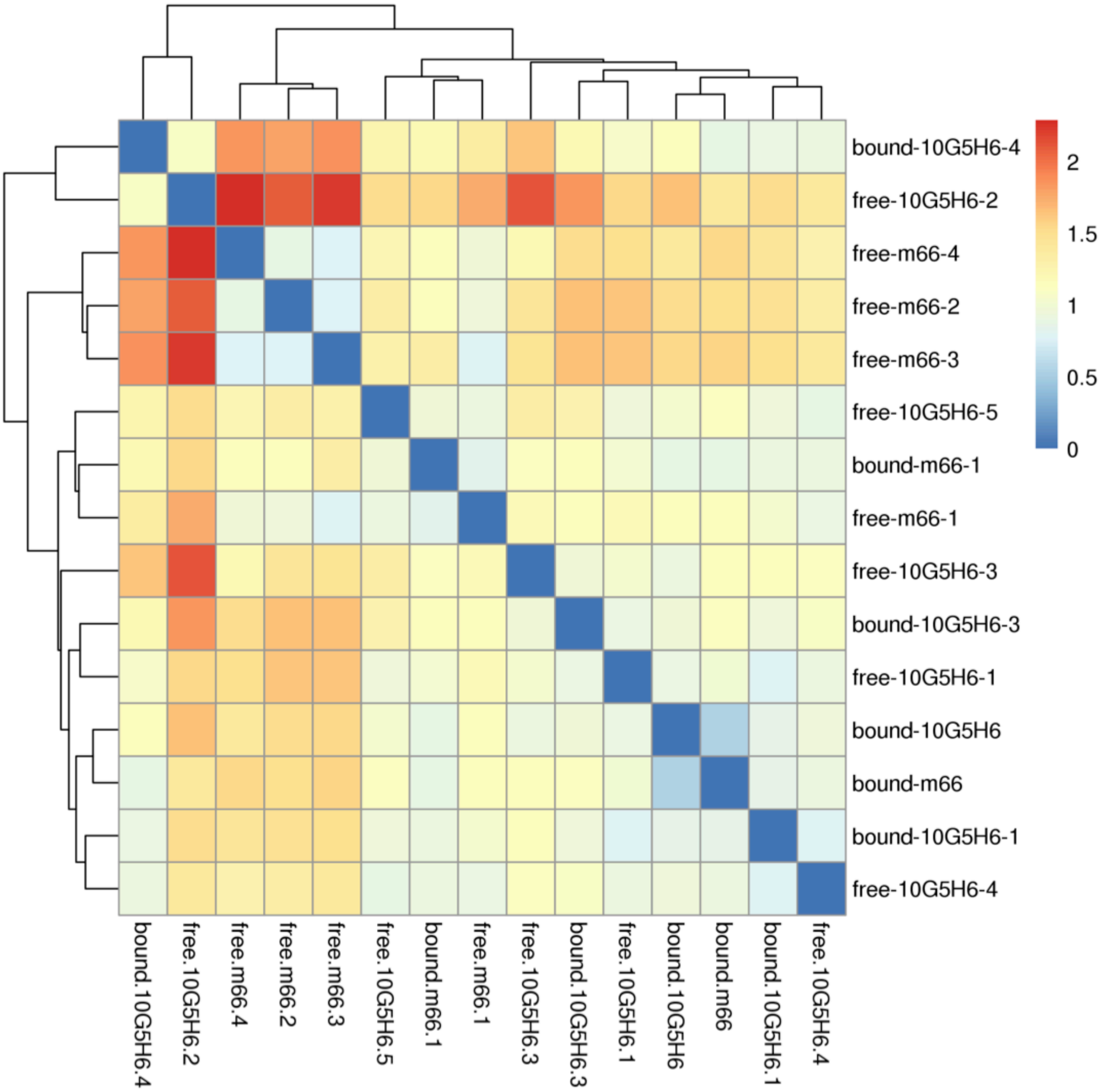

Figure S6. Heatmap of RMSD between conformers and crystal structures of antibodies of *V_H5-51* origin. Pair wise structural comparison of crystal structures and all conformers (bound and free) obtained after clustering of antibodies of *V_H5-51* origin from human, plotted along X-axis and Y-axis. Names of all conformers end with a number to represent the clusters. Crystal structures are named as bound-m66 (PDB ID: 4NRX) and bound-10G5H6 (PDB ID: 4HWB). Result is shown as a measure of RMSD in a gradient from blue (low) to red (high).

Table S3. H-bond (above 30 % occupancy across trajectory) of antibody complexes of mouse V_H*1-84* lineage

Molecule	Acceptor	DonorH	Donor	Percent occupancy
Ab-CD28	antigen		antibody	
	GLU_316@OE2	TYR_205@HH	TYR_205@OH	41
	GLU_316@OE1	TYR_205@HH	TYR_205@OH	38
	GLU_316@CD	TYR_205@HH	TYR_205@OH	49
	antibody		antigen	
	GLY_206@O	TYR_280@HH	TYR_280@OH	49
Ab-DNA	antigen		antibody	
	DT5_1@O4	ASN_147@HD21	ASN_147@ND2	66
	DC3_2@OP2	HIE_33@HE2	HIE_33@NE2	58
	DC3_2@O2	GLY_212@H	GLY_212@N	55
	antibody		antigen	
	TYR_145@CD2	DT5_1@H3	DT5_1@N3	66
	GLY_98@O	DT5_1@HO5'	DT5_1@O5'	47
	GLY_98@O	DC3_2@H42	DC3_2@N4	42
Ab-uPAR	antigen		antibody	
	ASN_190@O	TRP_325@HE1	TRP_325@NE1	93
	GLY_217@O	TYR_308@HH	TYR_308@OH	93
	GLN_189@O	TYR_483@H	TYR_483@N	88
	ASN_220@O	ASN_332@HD21	ASN_332@ND2	66
	GLN_189@OE1	ASN_482@HD22	ASN_482@ND2	54
	GLN_221@OE1	ASN_332@HD22	ASN_332@ND2	34
	antibody		antigen	
	PHE_327@CE2	ASN_220@HD21	ASN_220@ND2	86
	TYR_308@OH	ASN_220@HD22	ASN_220@ND2	83
	ASN_332@OD1	ARG_192@HH21	ARG_192@NH2	81
	THR_333@O	ARG_192@HH22	ARG_192@NH2	74
	PHE_327@CD2	ASN_220@HD21	ASN_220@ND2	59
	GLU_334@OE1	ARG_192@HH12	ARG_192@NH1	56
	GLU_334@OE2	ARG_192@HH12	ARG_192@NH1	49
	ASP_331@O	THR_267@HG1	THR_267@OG1	44
	ASN_482@OD1	GLN_189@H	GLN_189@N	35

Table S4. H-bond (above 30 % occupancy across trajectory) of antibody complexes of human V_H*5-51* lineage

Molecule	Acceptor	DonorH	Donor	Percent occupancy
Ab-MPER	antigen		antibody	
	LEU_240@O	TRP_33@HE1	TRP_33@NE1	73
	ASP_241@OD2	THR_111@H	THR_111@N	52
	ASP_241@OD2	ARG_110@HA	ARG_110@CA	42
	GLU_236@CD	SER_220@HG	SER_220@OG	38
	LEU_246@C	ARG_110@HH11	ARG_110@NH1	34
	LEU_238@O	ARG_110@HH21	ARG_110@NH2	34
	ASP_241@OD2	THR_111@HG1	THR_111@OG1	31
	GLU_236@OE1	THR_221@H	THR_221@N	31
	antibody		antigen	
	TYR_107@O	SER_245@H	SER_245@N	74
	SER_31@O	TRP_243@HE1	TRP_243@NE1	52
Ab-IL13	antigen		antibody	
	SER_30@O	ASN_213@HD21	ASN_213@ND2	81
	VAL_49@O	SER_280@HG	SER_280@OG	72
	GLN_53@OE1	ASN_213@H	ASN_213@N	59
	LEU_33@O	ASN_213@HD22	ASN_213@ND2	52
	THR_91@O	TYR_259@HH	TYR_259@OH	48
	HIE_47@ND1	ARG_281@H	ARG_281@N	38
	antibody		antigen	
	TYR_318@OH	LYS_93@H	LYS_93@N	94
	ASP_167@OD1	ARG_31@HE	ARG_31@NE	61
	ASP_167@OD2	ARG_31@HE	ARG_31@NE	61
	ASP_167@OD2	ARG_31@HH21	ARG_31@NH2	56
	ASP_167@OD1	ARG_31@HH21	ARG_31@NH2	52
	ASN_213@OD1	GLN_53@HE21	GLN_53@NE2	49
	SER_143@O	GLN_53@HE22	GLN_53@NE2	44
	GLY_166@O	ASN_27@HD22	ASN_27@ND2	35
	ASN_213@CG	GLN_53@HE21	GLN_53@NE2	32
